# The New Theories of Tuberculosis

**Published:** 1856-04

**Authors:** 


### EDITORIAL DEPARTMENT.

*The New Theories of Tuberculosis.*—The perusal of an article by Dr. B. H. Washington, of Hannibal, Mo., published in the Nashville Journal of Medicine and Surgery, reminds us that we have, perhaps, been negligent of our duty as a journalist, in not insisting sufficiently upon the value of the new ideas of the pathology and treatment of consumption, which are now usurping previous notions.

The contrast between the two ideas of treatment is a marked one. The venesection, the emetic, the blister, the iodine inhalation, the careful protection from air and from exertion, the abstinence from animal food and from stimulating drinks incident to our former ideas, have given place to active exercise, to fat meats and hearty diet, to vinous and alcoholic stimulants, and to what would once have been deemed reckless exposure to vicissitudes of weather.

"I've been bleeding from the lungs since I saw you!" said a medical friend as he entered our office, and in reply to our inquiry as to what he had done for it, he answered coolly, "Drinking whisky punch!" He followed up this pleasant remedy in moderation, and, so far as we know, has had no return of his attack.

In another instance a friend, also a medical man, bled profusely in the city of New York, when just upon the eve of an Atlantic voyage. Feeble and emaciated, he insisted on accomplishing his purpose, and sailed for London. He reached there in a very much improved condition, but was again attacked by alarming bleedings. He kept his bed for a fortnight, taking little medicine save morphine. As soon as his strength would permit, he started on his return voyage, as medical officer of a full emigrant ship; though still in a very exhausted condition. The faithful performance of his duty in enforcing cleanliness in the steerage, and driving the emigrants daily on deck during a long, rough passage, required a great deal of physical exertion and exposure, with constant occupation of mind. When he reached New York again, he was in unusually good health and flesh, and had the satisfaction of landing one more passenger than he took on board, no deaths, and one birth having occurred on the passage.

In still another, a gentleman who had had cough and purulent expectoration for some months, which had gradually developed the sequelæ of night-sweats and emaciation, came under our care. He presented, on physical exploration, marked dullness over the left infra-clavicular region, with a harsh and rough respiratory murmur. During a period of two years, since that time, he has traveled much, been extremely reckless of exposure, and drank alcoholic liquors in moderation. His diet has been generous, fat food being particularly enjoined. We saw him lately looking as robust and well as ever.

We could multiply cases. Even in those far advanced in disease, we have never failed to witness more or less improvement as the result of this regimen. If it is the most promising of cure to the curable patient, it is also the most conducive to comfort in the incurable. We do not intend to discuss the pathology of the disease, or the rationale of the treatment in this hasty sketch. We wish only to declare our full conviction of the merits of the tonic treatment.

The term *exercise*, in this connection, does not mean a gentle ride in a carriage on a pleasant day: it means *hardship*, positive hard work, involving fatigue and consequent good appetite, easy digestion and sound sleep. Neither should the patient avoid exposure to vicissitudes of weather at the expense of his digestive organs. It is far better to get wet in a storm, than to sit all day by the coal-grate, and get a headache and loss of appetite thereby. It is infinitely better to take cold than to grow dyspeptic.

We have not stated this too strongly. We are perfectly aware that, aside from the few cases mentioned, we have not advanced an argument in favor of the treatment. But we have stated, concisely, what the treatment is, and what may be expected from it; and there we leave it to the consideration of our readers.

